# Steady-state EEG captures how elementary classroom instruction drives plasticity for novel visual words

**DOI:** 10.1038/s41539-025-00371-w

**Published:** 2025-11-20

**Authors:** Fang Wang, Elizabeth Y. Toomarian, Radhika S. Gosavi, Blair Kaneshiro, Anthony M. Norcia, Bruce D. McCandliss

**Affiliations:** 1 Graduate School of Education, Stanford University, Stanford, CA USA; 2 Synapse School, Menlo Park, CA USA; 3 Department of Psychology, Stanford University, Stanford, CA USA; 4 Wu Tsai Neurosciences Institute, Stanford University, Stanford, CA USA

**Keywords:** Education, Cognitive neuroscience, Education, Psychology

## Abstract

Early readers encounter thousands of printed words in children’s books. The frequency with which they see each word shapes both neural and behavioral responses. Teachers also introduce novel written words through short, intensive learning experiences. Here we combined steady-state visual evoked potentials (SSVEP), corpus-based word frequency counts, and a novel two-week classroom “learning sprint” to examine and compare these two forms of experience-dependent plasticity. Cortical responses at 4 Hz to contrasts between real words of varying frequency (high: on average 1000 per million; medium: on average 200 per million) and pseudowords were sensitive to corpus-based frequency estimates—marking the first such finding using SSVEP. Strikingly, newly acquired low-frequency words (<1 per million)—taught in a child’s own classroom versus counterbalanced words taught in two other classrooms—elicited cortical responses nearly identical to those evoked by high-frequency words versus pseudowords. Furthermore, 1 Hz responses to new vocabulary learning was linked to individual differences in reading skills, including word decoding and rapid automatic naming. Together, these findings highlight the causal impact of authentic instruction and the value of neuroscience-informed methods in education research.

## Introduction

Vocabulary plays a pivotal role in both communication and academic success. Hence, it is important to understand the neural mechanisms underlying vocabulary learning, especially in early readers. Children’s vocabularies grow by thousands of words each year, both through incidental, cumulative encounters with words in books and verbal contexts, as well as through explicit, systematic teaching in school, where the pronunciation and meaning of novel words are directly taught to children^1^.

The most established method for investigating the impact of readers’ cumulative experiences with words over their reading lifespan is corpus-based word frequency. Estimates of word frequency are typically based on the number of occurrences within a large sample of commonly used children’s books. Across a range of measures assessing fluency and accuracy, high-frequency words (HFW) are processed more efficiently than low-frequency words^2^, a phenomenon well known as the word frequency effect (for a review, see ref. ^3^) and is evident in both early readers and adults. Our recent work has shown that the brain signals of early readers (i.e., kindergartners to second graders) in response to visual word forms are significantly influenced by their cumulative prior experience with the specific visual word. Specifically, neural circuits exhibit stronger activation to familiar, HFW compared to unfamiliar pseudowords^4^.

Educational neuroscience, an interdisciplinary field addressing questions neither cognitive neuroscience nor education research can address alone, seeks to investigate how instructional practices shape experience-dependent brain plasticity in learning^5^, providing novel insights into how educators can design learning experiences that effectively support vocabulary growth and broader academic development. As educational neuroscience becomes more situated within school settings, it becomes possible to investigate not only how neural responses reflect cumulative exposure to visual words, but also how deliberate, week-to-week, explicit efforts to teach new visual words in school drive changes in neural responses to these new words. Research in this direction has the potential to inform teaching methods, curriculum development, and educational policies.

Research on the neural mechanisms of new vocabulary learning has been constrained by its primary reliance on laboratory-based efforts. Such laboratory studies investigate how learning experiences impact brain responses by introducing artificially constructed vocabulary items, typically presented through regimented protocols over a short series of training sessions, delivered by computer to individual learners in a controlled lab setting^6,7^. Such approaches limit the ecological validity of studying classroom learning dynamics, particularly in terms of how educational experiences shape the formation of novel neural representations for newly acquired vocabulary. To provide more relevant insights into how educational practices can be optimized to improve learning outcomes, class-based naturalistic studies of teaching and learning in schools offer greater relevance than generic, computer-based learning in a laboratory.

The current study was specifically motivated by this distinction between *generic* and *educational* forms of experience-dependent brain plasticity. Neural responses to words are known to be influenced by cumulative exposure to specific word tokens^8^. Such experience can be captured through corpus-based measures of word frequency, as well as through more controlled manipulations in which teachers intentionally embed new vocabulary into their lessons. Conducting this work in an EEG-equipped school setting^9^ allowed us to investigate not only cumulative, corpus-based effects of word frequency but also the immediate impact of embedding novel words into authentic classroom instruction over a two-week period, using counterbalanced teacher-word assignments. This design provided a unique opportunity to test, for the first time, how direct, short-term classroom learning shapes the neurocognitive processes that support the acquisition of new vocabulary in early readers, and to compare such learning with more generic word-learning experiences captured by corpus-based approaches. Specifically, we asked whether newly learned words in the classroom elicit neural responses similar to or different from words children encountered in books in a less constrained context, with learning experiences presumably accumulating over a much longer period of time. To this end, we contrasted HFW with pseudowords, medium-frequency words (MFW) with pseudowords, and very low-frequency words (fewer than one token per million) that were either embedded in the child’s own classroom lessons for two weeks or instead introduced in a peer classroom (counterbalanced). Based on previous literature^10^, we hypothesized that: (1) brain responses (e.g., topography and amplitude) to the contrast between newly learned words in a child’s own classroom and words introduced only in a peer’s classroom (counterbalanced) were expected to resemble those observed for the contrast between HFW and pseudowords; and (2) HFW would elicit a higher amplitude than MFW when compared to pseudoword controls. Our approach builds on previous electrophysiological work with Steady-State Visual Evoked Potential (SSVEP) paradigms, which have proven to be highly sensitive to lexical experience-dependent learning in early readers^4,11^.

The SSVEP paradigm typically elicits a neural response between two categories of stimuli presented at two distinct, experimentally defined periodic rates. For example, word oddballs may be presented at 1 Hz (once per second), embedded within a stream of pseudowords presented at 3 Hz (three times per second), forming a 1 Hz oddball and 3 Hz base stimulation frequency. This temporally periodic presentation elicits periodic neural responses at the predefined stimulation frequencies and their harmonics (i.e., all integer multiples of the stimulus frequency). One of the most attractive features of this approach for measuring cortical responses is that it has been shown to elicit much higher signal-to-noise ratio (SNR) responses than other EEG paradigms^12^. In fact, these benefits are so large, that the SSVEP paradigm enables reliable measurement of neural responses for each condition with just 10 trials of 10 s each (i.e., 100 s of data^4^). This approach offers practical advantages that complement traditional Event-Related Potential (ERP) methods. It enables the simultaneous assessment of word learning effects and word frequency sensitivity within a single, time-efficient session—particularly valuable when working with young children in school-based settings. The brief and engaging format helps sustain children’s attention and allows the entire session to fit within blocks of a typical school-day schedule, an important consideration for maintaining classroom routines.

Taken together, this approach for quantifying the impact of different forms of learning experiences on cortical responses to specific word tokens enabled us to carry out this entire study, including EEG data collection, within an elementary school setting, seamlessly integrating the protocol into the natural rhythms and demands of the school day. This feat was facilitated by an existing Research-Practice Partnership (RPP) between our university research group and a local school^9^; Researchers and practitioners co-designed the study over the course of nearly a year. Learning strategies and activities reflected teachers’ authentic daily practices. During the two-week “learning sprint”, teachers led their class in learning a list of uncommon vocabulary words for ~15 min per school day. Moreover, students were not only participants but also played an active role in the research process through constant interactions with researchers and teachers. Cognitive processes underlying new word learning were assessed using EEG, recorded in a dedicated laboratory inside the school^9^. The study’s design, which randomized the selection of words for teaching while reserving others as controls, is positioned to investigate a causal link between specific learning activities and neural changes in word representations.

Class-based, naturalistic learning studies such as the current investigation provide a unique extension of existing knowledge on children’s vocabulary learning. Such studies may also shed new light on theories of word reading development, specifically by highlighting the causal role of classroom learning experiences intentionally scaffolded by teachers around specific vocabulary. These studies may also yield practical advantages, such as insights for educational interventions and activities that promote efficient vocabulary acquisition and improve reading fluency.

## Results

### Evidence for new word learning at the level of behavioral responses

**Fig. 1:**
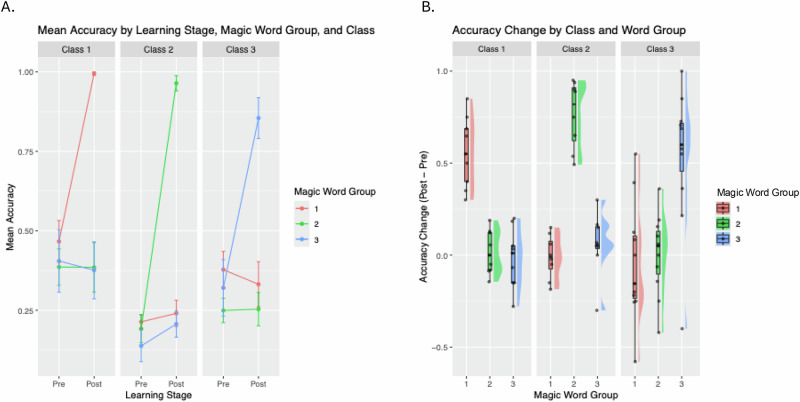
New word learning at behavioral level: Lexical decision task performance. **A** Mean accuracy of behavioral responses to words that children learned in their own class and those they did not learn (assigned to the other two classes), before and after the two-week learning sprint; **B** Mean accuracy changes (Post-Pre learning) of behavioral responses to words that children learned in their own class and those they did not learn in the other two classes. Children’s accuracy changed significantly for words they learned but not for those they did not learn. There was no significant difference across the three classes in the word learning effect.

Within each of the three classrooms, mean accuracy changed significantly (all *p* < 0.001) for the words students learned in their own class, but not for words in other classrooms. A one-way ANOVA showed no significant difference across the three classes (*F*(2, 83) = 0.86, *p* = 0.43) in the word learning effect. The range, mean, and standard deviation (M ± SD) of accuracy from the behavioral word familiarity task (modeled after common lexical decision tasks used with early readers) for each class and each word group (one group learned by the student and two groups not learned) are summarized in Fig. 1 and Table 1.

**Table 1:** Lexical decision task performance (accuracy) pre- and post-learning across three classes

Class	Pre		Post	
	Range	Mean(SD)	Range	Mean(SD)
Class 1	0.15–0.7	0.47 (0.27)	0.95–1	0.99 (0.02)
Class 2	0.05–0.35	0.19 (0.15)	0.8–1	0.97 (0.35)
Class 3	0–0.79	0.28 (0.28)	0.5–1	0.91 (0.39)

Range, mean, and standard deviation (SD) of response accuracy for the magic word learning task are shown for each class.

### Evidence for new word learning at the level of cortical responses

Results from base frequencies generally reflect visual processing common to all stimulus classes in each trial, while the oddball results focus specifically on the contrasts between two stimulus classes. Given our focus here on contrasts between stimulus classes, results from analysis of base frequencies will be reported in the supplement.

**Fig. 2:**
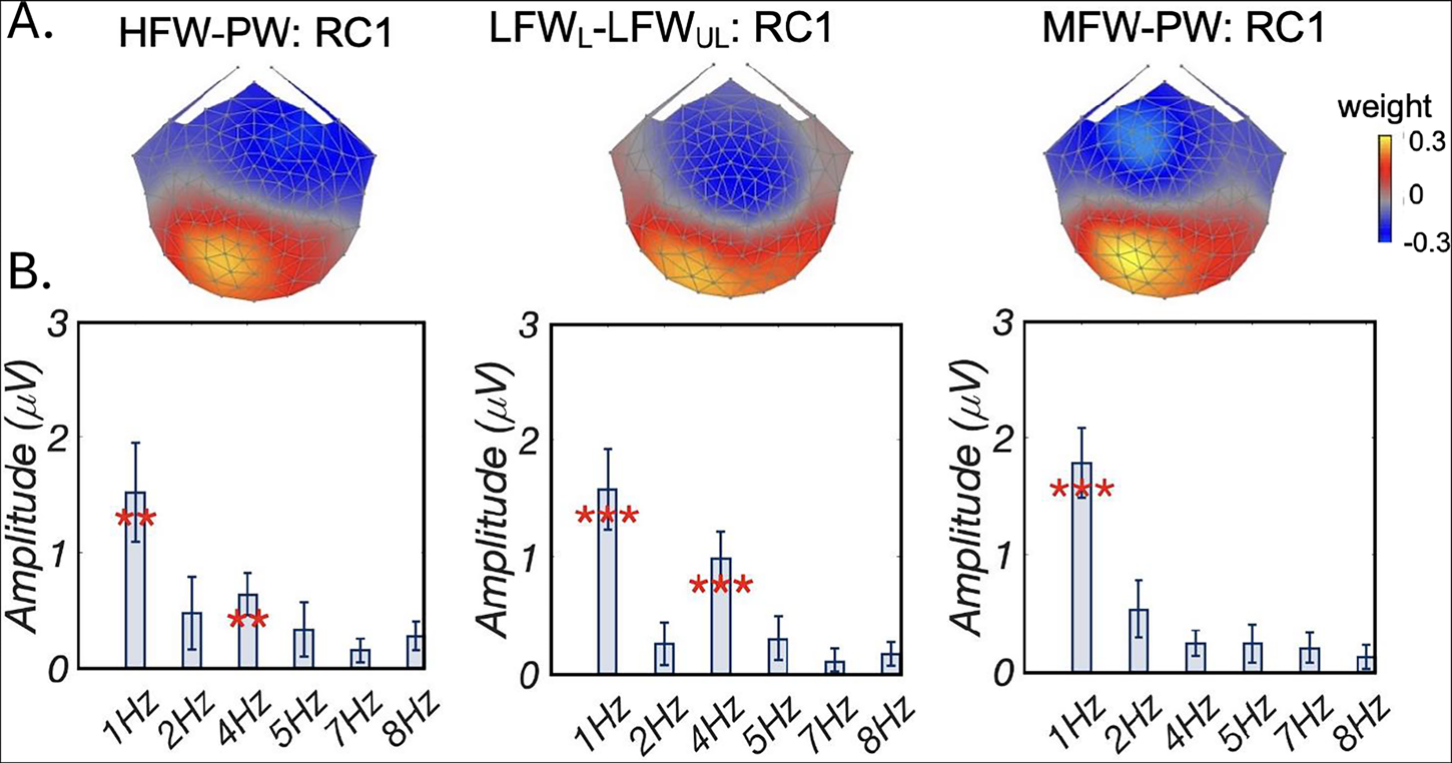
Comparison of brain responses to contrasts of HFW-PW, LFW_Learned_- LFW_Unlearned_, and MFW-PW. **A** Topographic visualizations of the spatial filters for the first reliable component (RC1) in three conditions; **B** Amplitude of responses at each harmonic of the oddball. Only neural responses at 1 Hz and 4 Hz were significant. At 1 Hz, comparable response amplitudes were found across three conditions. At 4 Hz, HFW-PW and LFW_Learned_- LFW_Unlearned_ evoked significantly higher (both *p*_*FDR*_ < 0.01) amplitude than MFW-PW; no significant difference was found between HFW-PW and LFW_Learned_-LFW_Unlearned_. **p_*FDR*_ < 0.01, ***p_*FDR*_ < 0.001. Error bars are ±1 SEM. HFW-PW high frequency words-pseudowords, LFW_Learned_- LFW_Unlearned_ learned low frequency words-unlearned low frequency words, MFW-PW medium frequency words-pseudowords, FDR fase discovery rate.

For responses to all three stimulus contrasts/conditions, only the first, maximally reliable component (RC1) contained significant signals (i.e., significant permutation test *p*-values and at least one significant harmonic). Results are summarized in Fig. 2.

Figure 2A displays topographic visualizations of the spatial filter for RC1 in three conditions. RC1 topographies were highly correlated (*r* > .81) across the three conditions and maximal over left occipito-temporal area. The bar plots in Fig. 2B present amplitudes, statistically significant responses were observed at first and fourth harmonics (all *p*_*FDR*_ < 0.01, corrected for 6 comparisons) for HFW-PW and LFW_Learned_- LFW_Unlearned_; but only at the first harmonic (*p*_*FDR*_ < 0.001, corrected for 6 comparisons) in MFW-PW.

One-way ANOVA of response amplitudes at the first harmonic (1 Hz) did not show a main effect of condition (*F*(27, 54) = 0.09, *p* = 0.92, ${\eta }_{p}^{2}=0.64$), indicating comparable response amplitudes at the first harmonic across three conditions. However, one-way ANOVA at the fourth harmonic (4 Hz) showed a significant condition effect (*F*(27, 54) = 5.17, *p* < 0.01, ${\eta }_{p}^{2}=0.49$). To contextualize the effect size results, our sample size and design provide 80% power to detect partial ${\eta }_{p}^{2}$ values as low as 0.28 (Cohen’s *f* = 0.98), as calculated using the *pwr* package in *R*. Post-hoc paired *t* tests (one tailed) showed that response amplitudes at 4 Hz in conditions HFW-PW and LFW_Learned_- LFW_Unlearned_ were significantly larger (both *p*_*FDR*_ < 0.01, corrected for three comparisons) than that in MFW-PW condition. No significant difference (*t*(27) = 1.60, *p* = 0.12) was found between HFW-PW and LFW_Learned_- LFW_Unlearned_. These results indicated similar responses between HFW and LFW_L_.

### Individual differences in new vocabulary learning

**Fig. 3:**
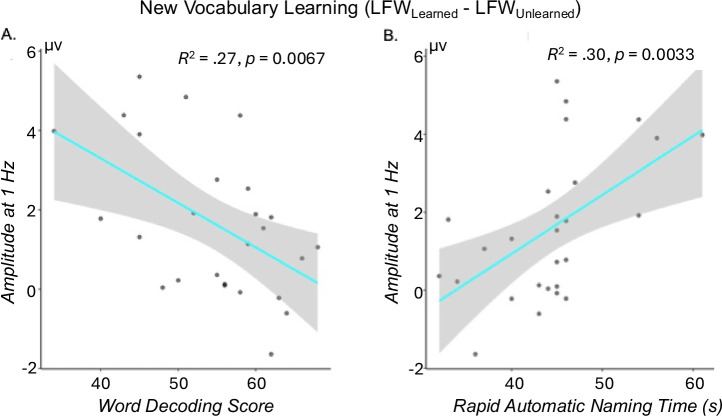
Statistically significant correlations between reading and new vocabulary learning, after outlier removal. **A** Correlation between amplitudes at 1 Hz for LFW_Learned_- LFW_Unlearned_ and word decoding ability (WJ-LWID); **B** Correlation between amplitude at 1 Hz for LFW_Learned_- LFW_Unlearned_ and Rapid Automatic Naming (RAN, color). Children with smaller response amplitudes to new vocabulary learning had better reading performance in word decoding and rapid automatic naming. No significant relations were found for the other two contrasts: HFW-PW and MFW-PW. HFW high-frequency words, MFW medium-frequency words, LFW low-frequency words, PW pesudowords.

For the contrast of LFW_Learned_-LFW_Unlearned_, we found significant correlations between EEG amplitudes at 1 Hz and both word decoding ability (measured by Woodcock–Johnson letter word identification, WJ-LWID, *R*^2^ = .27, *p* < 0.01) and rapid naming ability for color (RANcolor, *R*^2^ = .3, *p*_*FDR*_ < 0.01, Fig. 3). Children with smaller response amplitudes to new vocabulary learning had better reading performance (faster RAN and better word decoding). No significant correlations were found for amplitudes at 4 Hz (all *R*^2^ < .1, *p* > 0.11) in this contrast. For the contrasts of HFW-PW and MFW-PW, no significant correlations were found between response amplitudes and reading scores (all *R*^2^ < .12, *p* > 0.1).

## Discussion

We examined experience-dependent effects in cortical responses to specific word tokens in early readers using two complementary approaches. The first approach drew on the predominant corpus-based method, selecting high- and medium-frequency words from children’s book corpora and matching them to pseudowords on sublexical properties, isolating differences arising from children’s prior exposure during typical reading development. The second was more novel: In collaboration with teachers, we co-created a classroom-based “learning sprint” in which words were prospectively embedded into two weeks of instruction, allowing us to test causal links between experience and cortical responses. Together, these corpus- and classroom-based approaches provide converging evidence for how specific word-learning experiences shape cortical processing, advancing the goals of Educational Neuroscience to connect practice with changes in brain function.

Using learning sprint designs to embed learning manipulations within authentic classrooms extends the implications of previous lab-based studies of experience-dependent effects for visual words. RCA of learned low-frequency words in the LFW_Learned_-LFW_Unlearned_ contrast generated a component that is distributed over the left occipito-temporal (OT) electrodes. This aligns with prior studies showing robust training effects over the left OT for whole-word or single-letter processing after short-term training^13–17^. However, most training studies to date used stimuli from a novel language system^13,14^ or an artificial script^15–17^ in controlled lab settings, which limits the generalization to actual pedagogical practice and real vocabulary learning. For instance, with artificial grapheme-phoneme correspondence training, Pleisch and colleagues found more pronounced activation for trained than untrained *false-font* characters in the left OT^17^. The current study extends previous work by demonstrating a learning effect for real 5-letter words, and crucially, in a natural school context instead of a laboratory-based training experiments.

The present study additionally extends previous studies by comparing discriminative responses between learned real words in a child’s own classroom vs. counterbalanced real words, rather than symbols or pseudowords. The words learned in a child’s own classroom and the counterbalanced words from other classrooms were well-matched in unigram, bigram, and trigram frequencies, as well as orthographic neighborhood sizes (see detailed statistics in Section “Stimuli”). Such contrasts point towards a learning effect at the lexical level, rather than mere visual familiarity—since the similar amplitude and topography observed for learned low-frequency and HFW argue against a purely familiarity-based account—or mixed effects of learning at several levels of analysis (e.g., letter forms, sublexical and lexical information) that were captured in prior learned word vs. symbol contrasts. Our results most likely demonstrate the development of lexical representation after short-term of learning and critically extend previous knowledge about the relationship between word characteristics (e.g., word frequency) and lexical representation’s retrieval.

Although this study focuses on how changes in experience with particular words influence SSVEP oddball responses to those words versus more novel forms, our previous work has examined how oddball response amplitudes relate to individual differences in reading skill at this age. In general, these earlier studies found little to no association between responses to frequent words versus pseudowords in both cross-sectional^4^ and longitudinal^11^ samples of early readers in first and second grades. Here, we replicate this pattern: No significant correlations were observed between reading skill and oddball amplitudes in the HFW-PW and MFW-PW conditions. When examining the 1 Hz experience-based oscillation, however, we uncovered a significant brain-behavior correlation between oddball response amplitude and individual differences in decoding skill. This raises the question of why our learning sprint approach was more sensitive to brain-behavior relationships than corpus-based or categorical contrasts between words and pseudowords. Several factors may be relevant. First, corpus-based frequency estimates provide only an indirect and stochastic reflection of a child’s unique lexical experiences, whereas the learning sprint directly aligns the experiences with the specific words in the learning condition. Second, differences may reflect effects of spaced (i.e., accumulated experience for high- and medium- frequency words from corpus) versus massed practice (i.e., two-week learning sprint). Third, individual variability may be amplified for low-frequency words learned over a short period compared to high- or medium-frequency words with more established familiarity. As the present design does not allow us to distinguish among these possibilities, we focus our discussion on the implications of the brain-behavior relationship observed within the learning sprint.

Specifically, children with relatively dysfluent decoding skills, as indicated by WJ-LWID scores, showed larger oddball responses to contrast between words they learned in their own classroom versus words from other classrooms (counterbalanced). Word decoding can refer to the mapping of letters and groups of letters within words to their corresponding sounds—a process that encompasses a number of lexical and sublexical components including phonological awareness and orthographic processing^18,19^. For typical readers, word decoding has been used as a self-teaching strategy that establishes new written words in memory^20^. However, many children with reading difficulties such as dyslexia have problems with word decoding, which in turn impacts the development of reading fluency^21,22^. Our findings underscore and extend previous evidence showing that decoding ability plays a crucial role in word recognition^23^ and reading acquisition^24^.

The correlation between SSVEP responses to newly learned vocabulary words and decoding ability was reinforced by its association with RAN. A task involving retrieval of associations between visual symbols and phonological codes (names), RAN predicts reading skills^25–27^, and impacts literacy acquisition in elementary school children across different languages^28,29^. Some researchers consider RAN as a microcircuit of the later-developing reading circuitry^30,31^. For instance^30^, suggested that RAN taps object-naming circuits in the left hemisphere that are recruited to form the basis of the child’s developing visual word recognition system^30^. Overall, correlation results in the current study provide further support for the idea that children rely on phonological decoding skills (the ability to sound out words/objects) to learn novel words.

Reading development involves a shift in how different levels of representations are recruited, from basic visual features to letter forms, sublexical letter combinations, and ultimately lexical items^32^. The negative correlation pattern between learning and phonological decoding may reflect differences in the balance between whole-word and sublexical processing across readers with varying skill levels. For example, a recent SSVEP study showed that sublexical orthographic sensitivity correlates positively with reading experience: More experienced readers exhibit larger SSVEP amplitudes when comparing orthographically legal versus illegal letter combinations, indicating greater sublexical tuning^11^.

Contrasting theoretical frameworks for word recognition may provide distinct interpretations of this correlation pattern. From a Dual-Route perspective^33^, these negative brain-behavior correlations can be understood as reflecting a race between a lexical route and a sublexical decoding route. The outcome of this race depends on both the strength of lexical knowledge and the efficiency of sublexical processes. For children with weaker sublexical skills, extensive practice with specific words can strengthen the lexical route, allowing it to win the race. Thus, larger learning effects in weaker decoders reflect a shift in balance between the two pathways. For unfamiliar words, the sublexical route dominates. Once a critical subset of words is learned well enough for lexical processing to outpace sublexical decoding, the brain undergoes a state shift in processing. The learning-sprint effect, and its relation to decoding, may therefore be explained as weaker decoders dynamically switching between lexical and sublexical pathways once per second, producing large oddball amplitudes, whereas stronger decoders consistently rely on the sublexical route, resulting in smaller oddball effects.

An alternative account comes from computational models of visual word recognition that begin without pre-specified mappings between orthography and other linguistic systems, and instead building phonological and semantic connections through learning^34^. These models do not posit distinct mechanisms for lexical versus sublexical knowledge; rather, they apply the same learning principles across multiple grain sizes, from letters and rimes to entire words^35^. Within this framework, less skilled readers initially rely on larger grain-size mappings (e.g., whole word orthographic codes mapped to whole word phonological codes), which accentuates differences between learned and novel words. When phonological networks are stronger, new words are represented as weighted mappings that capture recurring correspondences between sublexical orthographic and phonological codes, which results in known and unknown words being encoded in a more similar fashion. The SSVEP learning-sprint effect and its relation to decoding can be conceptualized in terms of similarity among orthographic-phonological weights across words. A poor decoder may produce large shifts in similarity space once per second, yielding a stronger 1 Hz signal. A skilled decoder, however, would produce relatively smaller shifts in similarity space when switching from learned words in a child’s own classroom to unlearned words (assigned to other classrooms), resulting in a weaker SSVEP oddball effect.

Although these contrasting frameworks cannot be directly distinguished by the current data, they create a generative tension that can motivate future studies and potentially inform different approaches to teaching. For instance, one implication of linking the SSVEP oddball effect to route switching in the Dual-Route Cascaded model is that no comparable effect should arise when contrasting well-formed versus poorly formed nonword strings. In contrast, the computational framework that posits word recognition and learning as operating equivalently across multiple grain sizes would predict oddball effects from contrasts between learned vs. novel words at various levels, for example, in a learning study focused on rime units.

In addition to revealing fresh insights into vocabulary learning in natural classroom settings, we found that both high- and medium-frequency words, in contrast with pseudowords, evoked activation over left occipito-temporal regions, which has been challenging in previous SSVEP studies^36,37^. We used a relatively explicit repetition detection task and slower presentation rates, potentially explaining this disparity. The task in our study may have directed more attention to linguistic aspects, unlike previous studies that may have focused more on implicit visual discrimination processes (e.g., color detection task in ref. ^37^). Additionally, the presentation rates used in previous studies (e.g., 1.2 Hz oddballs and 6 Hz base in ref. ^36^) might have been too high for early readers, impacting higher-order processes. Our findings of lexical cortical tuning to print over left OT in early readers might reflect either direct access to lexical representations as indicated by numerous fMRI studies showing that left vOT regions, especially anterior, are engaged in lexical processing^38,39^. Alternatively, it may also reflect indirect lexical access through phonological decoding or grapheme-phoneme mapping, as suggested by phonological mapping hypothesis^40^. As an example, a combined EEG/fMRI study showed that activation of VWFA is responsible for grapheme-to-phoneme conversion, especially during early reading phases when direct lexical mapping of orthographic information has yet to be built^38^.

A large number of studies have examined word frequency effects across different writing systems using different neuroimaging techniques^8,41,42^. Low-frequency words consistently elicit larger EEG/MEG responses and stronger fMRI activations over the left vOT compared to HFW, aligning with behavioral findings showing shorter reading time for HFW^43^.

The current study, for the first time, examined the word frequency effect using an SSVEP paradigm. We contrasted high- and medium-frequency words separately with pseudowords to examine lexical retrieval. In comparison with the MFW vs. pseudowords contrast, the HFW vs. pseudowords contrast elicited larger amplitude responses, particularly at the fourth harmonic (i.e., 4 Hz), suggesting facilitated access to lexical representations compared to MFW^44,45^. Responses at any one of the harmonics of the oddball presentation rate are indicative of neural discrimination of the alternating categories. The fact that not all of these harmonics reflect word frequency indicates that the oddball response is not due to a single process, as a single process would be expected to show the effect at all of its harmonics. Rather, the oddball response may represent a mixture of different underlying neural mechanisms, operating either in parallel or in sequence, only one of which is subject to word frequency. Although a direct contrast between high- and medium-frequency words was not included in our design, our goal was to isolate lexical access by comparing words of different frequency levels against well-matched non-lexical controls (pseudowords). This approach allowed us to assess relative lexical processing strength while minimizing confounds, and it serves as a benchmark for examining the extent to which newly vocabulary words are learned. Future studies incorporating a direct comparison between high- and medium-frequency words could further elucidate the frequency-dependent dynamics of lexical access observed here.

Our research team, in partnership with a local school, bridges education and neuroscience through collaborative studies in real-world educational settings. This enduring partnership facilitates classroom-based training studies, where educators co-design teaching activities and strategies. Additionally, the partnership includes a permanent EEG recording studio at the school, enabling direct investigation of how schooling impacts children’s brain development. The partner school is an independent, nonprofit transitional kindergarten-8th grade institution located in Northern California, enrolling approximately 300 students, with about 20% receiving financial assistance. Although the school’s student-to-teacher ratio is smaller than the average in public schools, the learning strategies and activities implemented in our studies reflect the teachers’ authentic daily practices. As such, we believe the instructional dynamics observed in this context are representative of effective, generalizable approaches that can inform broader educational settings. Pursuing research through this novel partnership approach also expanded the researchers’ ability to study naturalistic learning environments, gave teachers space to engage with research and reflect on their practice, and offered students a sense of agency in exploring how we learn new words and how those experiences shape the brain.

In summary, the present study revealed some converging evidence in support of a causal relationship between short-term classroom-based learning and word lexical representation-building via a RPP. This was demonstrated by the emergence, after two weeks of classroom-based learning, of discriminative responses between learned low-frequency words and unlearned low-frequency words, which are similar to the response pattern in the contrast of HFW vs. well-controlled pseudowords. In addition, we also found significant correlations between new vocabulary learning and reading skills, including word decoding and rapid automatic naming. The correlation results provide support for the notion that children rely on phonological decoding skills to learn novel words. Furthermore, responses to HFW (vs. pseudowords) were higher than that to MFW (vs. pseudowords), replicating the classic word frequency effect; however, as our design did not include a direct comparison between high- and medium-frequency words, this observation should be interpreted with caution. Taken together, the present results from a natural school context extend previous laboratory-based knowledge on learning of new vocabulary words. Research in this direction could be utilized to support effective learning approaches. We also highlight the RPP approach as an effective way to conduct neuroscience in ecologically valid educational settings, drawing more direct connections between education and neuroscience.

## Methods

### Participants

Forty-eight monolingual, native English-speaking children from three classes at an independent school participated in this two-week learning study. All participants completed a behavioral lexical decision task both before and after the learning sprint. Of these 48 participants, 30 children (ten from each class), with normal or corrected-to-normal vision and no known history of reading disabilities or neurodevelopmental/psychiatric disorders, participated in EEG sessions after the classroom learning sprint. Two participants (from different classes) were excluded due to data quality issues, resulting in *N* = 28 participants ranging in age from 6.75 to 8.89 years old (*m* = 7.69 years, *s* = 0.57 years, 14 males). The 28 participants whose EEG data were analyzed included 16 first graders (*m* = 7.32 years, *s* = 0.38 years, 9 males) and 12 s graders (*m* = 8.19 years, *s* = 0.38 years, 5 males). First and second graders were in the same mixed-grade class at the school. The study was conducted in the second half of the school year.

### General cognitive assessments

Each participant completed a 30-min individual behavioral session, on average two days (*s* = 3 days) after the EEG session. All children were tested for handedness (Edinburgh Handedness Inventory^46^), phonological awareness and rapid naming abilities using two sub-tests (Rapid Automatized Naming of letters and colors) of the Comprehensive Test of Phonological Processing, Second Edition (CTOPP-II^47^), word reading efficiency (Test of Word Reading Efficiency, Second Edition, TOWRE-2^48^), and word decoding ability using the sub-test of letter word identification (Woodcock–Johnson Tests of Achievement, Fourth Edition, WJ-IV,^49^. Results of the behavioral assessments are summarized in Table 2.

**Table 2:** Performance on behavioral assessments

Sex (female/male)	14/14	
Handedness (right/left)	26/2	
Age in years	6–8	7.7 (±0.6)
Test of word reading efficiency (TOWRE)	73–126	90.6 (±23.5)
Rapid automatic naming of colors (RANcolor)	22–51	34.0 (±7.3)
Rapid automatic naming of letters (RANletter)	14–41	21.0 (±5.5)
Word decoding ability (WJ-LWID)	32–88	53.9 (±12.3)

Values are range and mean(±*SD*). TOWRE: Number of real words and pronounceable nonwords read in 45 s. RAN: Time (s) used to quickly and accurately name all stimuli (e.g., letters or colors) on a test form. WJ-LWID: Number of correctly named letters and words. Note: All scores are grade-scaled scores.

### Study procedure and learning sprint

**Fig. 4:**
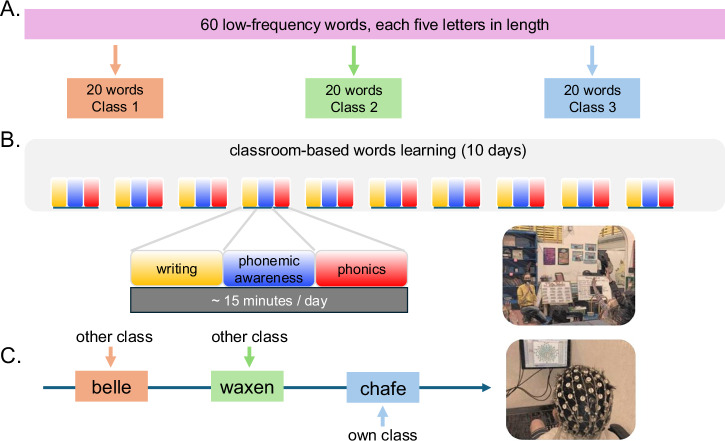
Study procedure. **A** Counterbalancing new vocabulary words. Researchers and teachers collaborated to design teaching strategies and create vocabulary lists. Sixty new low-frequency words were semi-randomly assigned to three classes (20 words per class); **B** Learning sprint. During a two-week learning sprint (15 min daily, 5 days per week), teaching activities focused on: (1) phonemic awareness (hear, identify, and manipulate phonemes in spoken words); (2) phonics (connect graphemes to phonemes in written words); and (3) writing (creating a handwritten representation of a word---whether by illustrating it, writing it from dictation, or using skywriting); **C** EEG recording at school. After completing their learning activities, participants individually visited the “brainwave recording studio” in their school for EEG recording. During the session, participants were shown words they had learned in their own class as well as words they had not learned because they were assigned to the other two classes.

Before initiating the classroom learning sprint, teachers were briefed on the general research idea, then engaged in collaborative brainstorming sessions with the research team to generate ideas and insights from their practice to assist with study design and implementation. Drawing from their collective feedback and existing teaching approaches, the research team assembled a tailored set of teaching strategies focusing on phonemic awareness, spelling, as well as handwriting, and developed a list of “magic” words for the sprint (see Section “Stimuli”). Following this, a research plan was collaboratively crafted to guide the study’s launch and initiate the learning sprint (Fig. 4A).

During the two-week classroom-based learning sprint, each teacher led their class in learning a unique list of 20 assigned new low-frequency vocabulary words (see Section “Stimuli”). Display boards, featuring laminated velcro cards labeled with the selected words, served as interactive tools for teachers to dynamically engage with their students. Participants engaged with the assigned word list for a minimum of 15–20 min per day, 5 school days per week, for two weeks total. A comprehensive tracker was created for each classroom, providing both teachers and students with a tangible, visual representation of their progress over the course of the learning sprint. Teachers implemented activities that were collaboratively designed in the earlier phase of the study, focusing specifically on: (1) phonemic awareness, (2) phonics, and/or (3) writing, encompassing activities such as vocabulary flashcards, spelling games, memory exercises, poetic exploration, Kahoot quizzes (an online game-based learning platform), creative writing, and more, which were all organic to the students’ learning environment and classroom practices (Fig. 4B). These parameters and activities were determined in partnership with school instructional leaders, intended to ensure instructional variety and emphasize the relationship between sounds and letters. This approach was specifically designed to help students attend to the internal structure of each word—particularly the grapheme-phoneme relationships—rather than relying on rote visual memorization. After each class finished their two-week learning sprint, participants individually visited the EEG “recording studio” lab in the school. Experimental sessions took place during the school day, allowing students to participate in research and expediently return to class (Fig. 4C).

### Stimuli

Sixty low-frequency words (LFW, <1 per million), each five letters in length, were chosen from the MRC psycholinguistic database^50^ and were semi-randomly divided into three lists, each consisting of 20 items. Unigram, bigram, trigram frequencies, number of phonemes and syllables, and orthographic neighborhood size were well matched across these three word lists (all *F*(2, 59) < 0.57, all *p* > 0.57), which were randomly assigned across the three classes. To enhance student engagement, we referred to these low-frequency words as “magic words”.

To better evaluate the degree of classroom-based word learning and compare short-term and long-term word learning, the study also involved HFW and MFW, all comprising five letters. The HFW (mean = 1006 per million, range 523–1821 per million) and MFW (mean = 221 per million, range 200–246 per million) were chosen from *The Educator’s Word Frequency Guide*^51^. Finally, five-letter pseudowords (PW) were included for the examination of lexical level representations compared to real words. PW were built on an item-by-item basis by shuffling letters across the set of high- and medium-frequency words used in the current study. PW were thus pronounceable and well-matched for orthographic properties (at both letter and structure level) of high- and medium-frequency words. Unigram, bigram, and trigram frequencies (all *t* < 1.98, all *p* > 0.05) and orthographic neighborhood sizes (*t*(69) = 1.29, *p* = 0.20) were matched between words and pseudowords. Detailed psycholinguistic characteristics of stimuli used in the study are summarized in Supplementary Table 1.

**Fig. 5:**
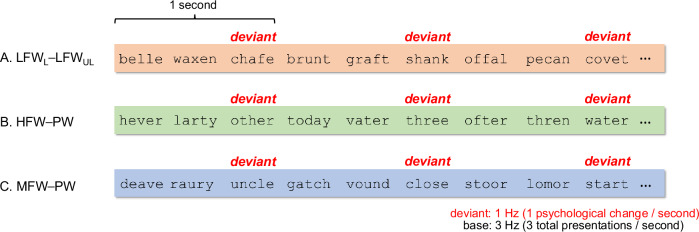
Experimental design. Examples of stimuli presented in the experiment. 1 Hz oddballs were embedded within a 3 Hz base stream in all three conditions. The first condition assessed processing differences between learned low-frequency words with well-matched unlearned low-frequency words (**A**. LFW_Learned_-LFW_Unlearned_). The second condition assessed processing differences between high-frequency words and well-matched pseudowords (**B**. HFW-PW). The third condition assessed processing differences between medium-frequency words and well-matched pseudowords (**C**. MFW-PW).

In all, the stimulus set comprised 20 HFW, 20 MFW, 60 LFW, and 80 PW, for 180 exemplars total. We investigated three experimental conditions as summarized in Fig. 5. In order to examine the effect of new word learning, in condition 1 (Fig. 5A), learned low-frequency word oddballs from a student’s own class were embedded in a stream of low-frequency words from the other two classes (LFW_Learned_-LFW_Unlearned_). To examine the extent to which the new vocabularies were learned in a short period of time, two other conditions were included: Condition 2 (Fig. 5B) involved HFW oddballs embedded in a stream of well-matched pseudowords (HFW-PW), while condition 3 (Fig. 5C) involved MFW oddballs embedded in a stream of well-matched pseudowords (MFW-PW). Condition orders were counterbalanced across participants and classes. All three conditions were presented at a base frequency of 3 Hz and a oddball frequency of 1 Hz. This means that three stimuli per second were presented at a constant rate (3 Hz) and—in condition 1 for example—the three stimuli presented in a given second always comprised one learned low-frequency word (1 Hz) followed by two low-frequency words (LFW_Unlearned_-LFW_Unlearned_-LFW_Learned_) introduced in other classrooms. Of note, to avoid potential confounding of stimuli repetition in SSVEP studies^52^, the pseudowords used in the HFW-PW condition were different from the pseudwords in the MFW-PW, which is why there are more pseudowords than word exemplars.

### Behavioral lexical decision task

All stimuli in the lexical decision task were the same as those used in the EEG session. Children were asked to decide whether a stimulus was a real word or not before and after the learning sprint. They pressed one button for a real word and one button for a pseudoword using their dominant hand. Mean accuracy in each class for each word group (one group a given student learned and two groups they did not learn) was calculated. One-way ANOVA with within-factor of class was computed on mean accuracy across three classes.

### EEG recording procedure

Prior to the EEG recording, a brief practice session was held to familiarize the participant with the experimental procedure and the repetition detection task. During the EEG recording, participants sat in a dimly lit room 1 m away from the computer monitor. Each stimulation sequence started with a blank screen, the duration of which was jittered between 1500 ms and 2500 ms.

Each trial comprised 12 s of stimulation, and each condition comprised 10 trials; thus, each participant completed 30 trials total across the three conditions. Participants were asked to press a button on an external response pad with their preferred hand when a stimulus (i.e., target) was repeated three times in a row. This repetition detection task was chosen based on pilot work showing that while children of this age can handle two-alternative forced-choice lexical decision tasks, yet the continuous performance format of our SSVEP design made such tasks more challenging. We therefore needed a task that young readers could sustain across repeated blocks within a single session, while still engaging lexical processing on each stimulus. Pilot results and previous findings^4,11^ confirmed that children persist and perform well on the repetition task across various stimuli (e.g., false fonts, orthographically legal/illegal strings, pseudowords, and words). Additionally, this task reliably elicits constrained EEG-SSVEP effects at both lexical and sublexical levels, even though it does not explicitly require orthographic, phonological, or semantic knowledge^4^.

**Fig. 6:**
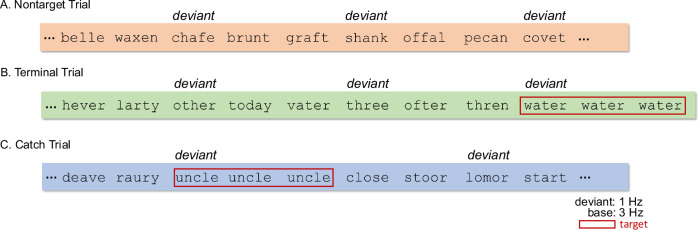
Example nontarget, terminal, and catch trials. Twelve trials were pseudorandomly presented for each condition, including four nontarget trials (**A**), four terminal trials (**B**), and two catch trials (**C**). Catch trials were excluded by design to minimize EEG contamination from motor responses associated with button presses. Data corresponding to the four terminal trials were still included because movements happened at the end of the trial, resulting in 8 out of 10 trials per condition per participant being used for analyses.

Among the 10 trials that were pseudorandomly presented for each condition, there were four “nontarget” trials which contained no repeated stimuli (Fig. 6A); four “terminal” trials in which repeated stimuli appeared at the end (Fig. 6B); and two “catch” trials with repeated stimuli randomly appearing elsewhere during the trial (Fig. 6C). Each terminal and catch trial contained only one target. Participants were given verbal feedback about their performance after the end of each trial. Due to excessive movements from the button press, each participant’s two catch trials were excluded in their entirety from further analysis. Data corresponding to the four terminal trials were still included because movements happened at the end of the trial. In all, 8 of 10 trials for each condition from each participant were used for analysis.

EEG data were collected using 128-sensor HydroCell arrays (MagstimEGI), Electrical Geodesics NetAmp300, and NetStation 5.4.2 software, while stimuli were presented via an in-house software. Data were acquired against Cz reference at a sampling rate of 500 Hz. Impedances were kept below 50 k*Ω*.

Overall, the entire EEG experiment took around 40 min per participant, including setup, practice, and breaks between trials and conditions.

### EEG preprocessing

EEG Recordings were bandpass filtered offline (0.3–50 Hz) using Net Station Waveform Tools. Subsequently, data was preprocessed and re-sampled to 420 Hz. Sensors for which more than 15% of samples from the sensor exceeded a 60 μV amplitude threshold were interpolated by the average value from six nearest neighboring sensors.

The continuous EEG data were then filtered with Recursive Least Squares (RLS) filters^53^ and re-referenced to average ref. ^54^. Segmented into 1-s epochs, data were screened for artifacts, excluding epochs with over 10% of data samples exceeding the noise threshold of 30 μV or any part surpassing the 60 μV blink threshold on a sensor-by-sensor basis. If an epoch exceeded the peak/blink threshold in more than 7 sensors, the entire epoch would be removed in all sensors. To mitigate initial transient responses, the first and last epochs of each 12-epoch, 12-s trial were omitted, leaving 10 epochs (i.e., 10 s) per trial for analysis.

The RLS filters were tuned to each of the analysis frequencies (base harmonics: 3 Hz, 6 Hz, 9 Hz; oddball harmonics, excluding base harmonics: 1 Hz, 2 Hz, 4 Hz, 5 Hz, 7 Hz, 8 Hz) and converted to the frequency domain by means of Fourier transform. Complex-valued Fourier coefficients were decomposed into real and imaginary coefficients for input to the spatial filtering computations of Reliable Components Analysis (RCA), as described below.

### Analysis of EEG data

We applied RCA^55^ to decompose the 128-sensor array into a set of *reliable components* (RCs) maximizing between-trials covariance. Unlike analysis methods that involve the preselection of sensors based on literature or SNR within a predefined cluster—an approach commonly used in ERP and SSVEP studies that might lead to a bias in reporting false positives^56^—RCA computes weighted linear combinations across the whole montage of sensors, yielding “reliable components” (RCs^57^). Compared to other spatial filtering approaches, such as Principal Component Analysis and Common Spatial Patterns, RCA produces component topographies that more closely resemble the underlying cortical sources (i.e., lead fields) generating the observed SSVEPs^55^. In addition, RCA achieves higher SNR with lower trial count by maximizing across-trial correlations (i.e., “reliability”) while minimizing noise power^55^.

SSVEP response phases remain constant across stimulations, rendering RC activations indicative of phase-locked activities. RCA operates on sensor-by-feature EEG data matrices, deriving linear spatial filters to maximize Pearson Product Moment Correlation Coefficients^58^ across trials. These filters transform data from sensor-by-feature to component-by-feature matrices, with each component representing data from a linear combination of sensors. RC scalp topographies are visualized using forward-model projections of spatial filter vectors^59^. Additional details on this spatial filtering technique are provided in Dmochowski et al.^55^.

Because signals after 10 Hz are very weak, we decided to use frequencies and harmonics within 10 Hz (i.e., base frequency and its harmonics: 3 Hz, 6 Hz, and 9 Hz; oddball frequency and its harmonics: 1 Hz, 2 Hz, 4 Hz, 5 Hz, 7 Hz, and 8 Hz, excluding base harmonics) for analyses.

For oddball analyses, which focus on neural processing disparities between oddballs and control (e.g., HFW vs. pseudowords), we typically analyze each condition separately to reveal potential similarities or differences underlying different stimulus contrasts. This encompassed real and imaginary Fourier coefficients at six harmonics, excluding base harmonics (1 Hz, 2 Hz, 4 Hz, 5 Hz, 7 Hz, and 8 Hz). Given that oddball responses are weaker than base responses - since they reflect contrast-specific rather than general visual processing - only one component typically reaches significance. Therefore, the analysis includes 6 harmonics × 1 component × 1 condition.

In contrast, base analyses examine signals at the base frequency and its harmonics (3 Hz, 6 Hz, and 9 Hz), which reflect general visual processing and test whether low-level stimulus features were well matched across conditions. We conduct RCA across all three conditions together, as the stimuli were carefully matched and we do not expect differences across conditions at the base frequency. The stronger base signal yields three significant components (RC1, RC2, and RC3; see Supplementary Fig. 1). Therefore, the base analyses include 3 harmonics × 3 components × 3 conditions (i.e., LFW_Learned_ vs. LFW_Unlearned_, HFW vs. PW, and MFW vs. PW).

We first assessed the significance of each component’s eigenvalue coefficient using permutation tests. This involved creating null distributions by generating 1000 surrogate data records with randomized phase spectra for each trial in sensor space before RCA computation. For more information, see ref. ^4^. Subsequently, we determined the significance of each harmonic within significant components using Hotelling’s two-sample *t*^2^ tests^60^. Data were projected through spatial filter vectors, then averaged across epochs and participants before statistical analysis. False Discovery Rate (FDR^61^) correction was applied for multiple comparisons. For base analyses, corrections were made for 27 comparisons (3 harmonics × 3 components × 3 conditions). For oddball analyses, corrections were applied for 6 comparisons (6 harmonics × 1 component) per condition. Statistically significant RCs, verified by permutation testing and containing significant amplitudes in at least one harmonic, were further analyzed and reported in the results.

For both the oddball and base analyses, we visualized the data in two ways. First, we present topographic maps for spatial filtering components. Second, we present bar plots of amplitudes (*μ*V) across harmonics, with significant responses (according to adjusted *p*_*FDR*_ values of Hotelling’s *t*^2^ tests of the complex data) indicated with asterisks.

### Assessing brain-reading relationships

Brain-behavior analyses were performed to assess individual variations in the relationship between EEG amplitude with reading scores. The reading scores analyzed were the participants’ grade-scaled scores of TOWRE, RANcolor, RANletter, and WJ. Here, we focused on brain responses to oddballs, which indicate discrimination responses between oddballs and control stimuli. For the HFW-PW and LFW_Learned_- LFW_Unlearned_ conditions, where the first and fourth harmonics (1 Hz and 4 Hz) were significant, linear correlations were performed on the averaged amplitude between these two significant harmonics. For MFW-PW, we performed linear correlations on projected amplitudes only at 1 Hz, which was the only significant harmonic.

If a significant correlation was found, influential data points were identified using Cook’s Distance^62^ and removed if they exceeded the CooksD 4/*n* threshold (where *n* is the total number of data points). Correlation analyses were then performed again to determine whether the significant relationship still held after the removal of influential data points. Brain response amplitudes to base stimuli were not used for brain-behavior analyses, since the corresponding neural activity related to processing of lower-level visual word features which was expected to be comparable across conditions.

### Ethics statement

This study was approved by the Institutional Review Board (Protocol 50742, PI: McCandliss) at Stanford University. Informed consent was obtained from participants’ parents/guardians prior to both classroom behavioral data collection and the EEG experimental session. At the beginning of the session, participants were given an overview of the experimental protocol and provided assent before the study began. As a token of appreciation, each participant received a small prize at the end of the session.

## Supplementary information

Supplementary Information

## Data Availability

Primary data are publicly available (https://osf.io/bwh67/).

## Code availability

All analysis scripts are publicly available (https://osf.io/bwh67/).
