# Supplementary material for: Steady-state EEG captures how elementary classroom instruction drives plasticity for novel visual words
Source: NPJ Sci Learn. 2025 Nov 20;10:83. doi: 10.1038/s41539-025-00371-w (PMC12635053; doi:10.1038/s41539-025-00371-w)
Supplement: Supplementary file 1 — Supplementary Information [file 41539_2025_371_MOESM1_ESM.pdf]

1 Supplementary Material

Base Analyses Results

We performed RCA on responses at the base frequency and its harmonics in order to investigate neural activity related to low-level visual processing and whether visual properties of different types of stimuli were well matched across conditions. Based on permutation test and Hotelling's t^2 test, we report spatial filter topographies and statistical analysis of the projected data for the first three RCs (RCs 1, 2, and 3) returned by RCA trained on three conditions together. Base RCA results are summarized in Supplementary Figure 1.

Supplementary Figure 1A displays topographic visualizations of the spatial filters for the first three reliable components that contained significant stimulus-driven activity (i.e., significant permutation test and at least one harmonic is significant). Supplementary Figure 1B shows summary plots of the responses in the 2D complex plane, with overlapping amplitudes (vector lengths) and phases (vector angles) between these three conditions. Supplementary Figure 1C presents projected amplitude (project sensor-space data through spatial filter) in bar plots and the derived latency estimations. The projected amplitude contained statistically significant responses in all three harmonics at each of the three RCs (all $p_{FDR} < 0.05$, corrected for 27 comparisons) for all three conditions.

RSS amplitude (across three significant harmonics) comparisons showed that there is no significant difference between conditions at each of three RCs (all $F(2, 83) < 0.35$, all $p > 0.70$). Latency estimation derived from phase slopes across significant harmonics were also similar between conditions (RC1: 147.99 ms, 147.29 ms, and 148.29 ms; RC2: 309.68 ms, 313.33 ms, and 314.38 ms; RC3: 84.28 ms, 93.65 ms, and 92.98 ms; for three conditions respectively).

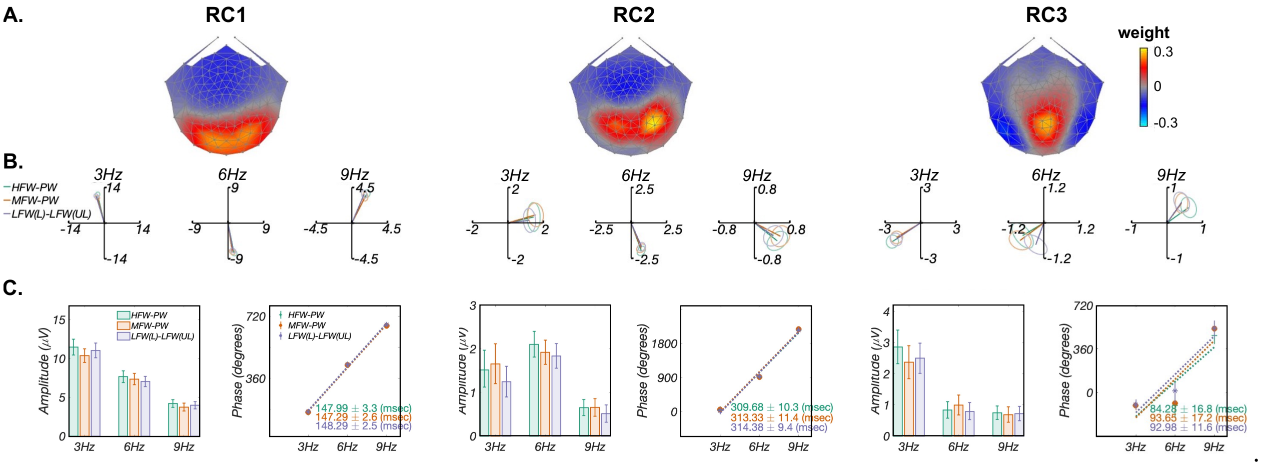

Supplementary Figure 1: **Base Analyses Results.** A: Topographic visualizations of the spatial filters for the first three components (RC1, RC2, and RC3); B: Response data of three conditions presented in the 2D complex plane, where amplitude information is represented as the length of the vectors, and phase information in the angle of the vector relative to 0 degrees (counterclockwise from 3 o'clock direction), ellipse indicates standard error of the mean (SEM); C: Comparison of projected amplitude and latency estimation across three conditions. The projected amplitude contained statistically significant responses in all three harmonics at each of the three RCs (all $p_{FDR} < 0.05$, corrected for 27 comparisons) for all three conditions. In addition, RSS response amplitudes across three harmonics did not differ significantly across three conditions for RCs 1-3 (all $F(2, 83) < 0.35$, all $p > 0.70$), latency estimations derived from phase slopes across harmonics were also similar across conditions.

Participants were equally engaged throughout the experiment

For the repetition detection task, means and standard deviations, $m(s)$, of *d'* were 1.04(0.38), 1.18(0.43), 1.14(0.40) for LFW_L– LFW_UL, HFW–PW, and MFW–PW, respectively. A one-way ANOVA showed that *d'* did not differ significantly across the three conditions ($F(2, 83) = 0.86, p = 0.43$). Thus, we conclude that participants were equally engaged throughout the three conditions of the experiment.

Psycholinguistic characteristics of stimuli

Condition	Stimuli	Unigram frequency		Bigram frequency		Trigram frequency		Orthographic neighbors		Word frequency	
		Range	Mean(SD)	Range	Mean(SD)	Range	Mean(SD)	Range	Mean(SD)	Range	Mean(SD)
Condition 1 (HFW-–PW)	**HFW**	4107–16252	9627(2748)	349–6515	2587(1592)	139–2003	1048(574)	0–9	5(3)	468–1821	831(386)
	**PW**	4495–15102	10224(2621)	308–5737	2602(1256)	95–2605	800(516)	0–10	4(3)	–	–
Condition 2 (MFW–PW)	**MFW**	6392–14934	10149(2161)	765–5308	1890(1034)	71–3704	588(771)	0–13	4(3)	200–246	220(14)
	**PW**	5782–13563	9847(2084)	568–3362	1710(752)	0.04–1267	342(322)	0–9	3(3)	–	–
Condition 3 (LFW_L– LFW_UL)	**LFW in Class 1**	4175–12555	8456(2529)	72–1572	801(423)	5–249	75(67)	0–6	2(2)	0.1–2	0.9(0.7)
	**LFW in Class 2**	3399–12526	7954(2296)	161–2272	816(581)	0.24–493	86(127)	0–8	2(3)	0.1–2	1(0.6)
	**LFW in Class 3**	2989–11711	7693(2028)	215–1576	682(360)	8–193	65(53)	0–7	2(2)	0.1–3	1(0.8)

Supplementary Table 1: **Psycholinguistic characteristics of stimuli used in the study.** The range, mean, and standard deviation (SD) of psycholinguistic characteristics are reported for HFW (high-frequency word), MFW (medium-frequency word), PW (pseudoword), and LFW (low-frequency words). HFW and PW in HFW–PW, MFW and PW in MFW–PW, and LFW used in three classes were all well-matched in uni-, bi-, and tri-gram frequencies, as well as orthographic neighborhood size.
